# Enhanced Perception for Autonomous Driving Using Semantic and Geometric Data Fusion

**DOI:** 10.3390/s22135061

**Published:** 2022-07-05

**Authors:** Horatiu Florea, Andra Petrovai, Ion Giosan, Florin Oniga, Robert Varga, Sergiu Nedevschi

**Affiliations:** Image Processing and Pattern Recognition Research Center, Computer Science Department, Technical University of Cluj-Napoca, 400114 Cluj-Napoca, Romania; horatiu.florea@cs.utcluj.ro (H.F.); andra.petrovai@cs.utcluj.ro (A.P.); ion.giosan@cs.utcluj.ro (I.G.); florin.oniga@cs.utcluj.ro (F.O.); robert.varga@cs.utcluj.ro (R.V.)

**Keywords:** autonomous driving, environment perception, low-level geometry and semantic fusion, semantic and instance segmentation, deep learning, 3D object detection

## Abstract

Environment perception remains one of the key tasks in autonomous driving for which solutions have yet to reach maturity. Multi-modal approaches benefit from the complementary physical properties specific to each sensor technology used, boosting overall performance. The added complexity brought on by data fusion processes is not trivial to solve, with design decisions heavily influencing the balance between quality and latency of the results. In this paper we present our novel real-time, 360${}^{\circ }$ enhanced perception component based on low-level fusion between geometry provided by the LiDAR-based 3D point clouds and semantic scene information obtained from multiple RGB cameras, of multiple types. This multi-modal, multi-sensor scheme enables better range coverage, improved detection and classification quality with increased robustness. Semantic, instance and panoptic segmentations of 2D data are computed using efficient deep-learning-based algorithms, while 3D point clouds are segmented using a fast, traditional voxel-based solution. Finally, the fusion obtained through point-to-image projection yields a semantically enhanced 3D point cloud that allows enhanced perception through 3D detection refinement and 3D object classification. The planning and control systems of the vehicle receives the individual sensors’ perception together with the enhanced one, as well as the semantically enhanced 3D points. The developed perception solutions are successfully integrated onto an autonomous vehicle software stack, as part of the UP-Drive project.

## 1. Introduction

Automation of individual transportation will bring major benefits once it becomes available in the mass market, such as increased safety on roads and higher mobility for the aging or disabled population. At the same time, electric automated vehicles have the potential to alleviate problems related to intensified urbanization by reducing traffic congestion with a more efficient coordination of vehicles, while at the same time reducing air pollution. Due to a lack of maturity in key technologies needed for the development of automated vehicles, the prospect of full vehicle automation remains a long-term vision.

**Figure 1:**
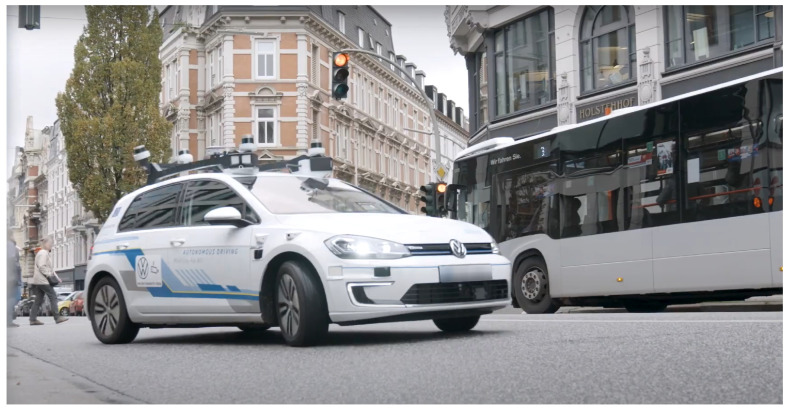
The UP-Drive demonstrator vehicle, based on a VW eGolf, navigates urban environments in fully automated mode at the completion of the project.

The European UP-Drive project [1], part of the Horizon 2020 program, set out to address these technological challenges by developing a fully automated electric vehicle capable of safely navigating complex urban environments (depicted in Figure 1). Its main use case covers the operation of a robot taxi, where passengers can be seamlessly transported from the pick-up point to their desired destination. Moreover, the vehicle is able to find empty parking spaces by leveraging a long-term semantic map shared between multiple vehicles in the fleet [2]. Creating such a system required the development of state-of-the-art key technologies that are essential to automated driving: robust 360${}^{\circ }$ perception, lifelong localization and mapping, scene understanding, risk assessment, planning and navigation.

The perception system of the automated vehicle, responsible for detecting and classifying the objects in the scene, has to fulfill the low (0) false negatives requirement, a more difficult one than the low false positives requirement specific to driving assistance systems. The solution for this problem is multiple redundancy, increased accuracy and robustness, along with 360${}^{\circ }$ coverage around the vehicle at the sensory and algorithmic levels. To achieve this goal, the UP-Drive vehicle was equipped with the area-view fish-eye camera system (4 RGB cameras) for surround view perception, a front narrow field of view camera for increased depth range and a suite of five 360${}^{\circ }$ LiDARs.

Our contributions are focused on the design and implementation of a perception-system architecture based on low-level fusion capable of delivering both classified 3D object detections as well as a semantically enriched 3D point cloud used for enhanced perception. Moreover, emphasis is placed on ensuring not only that the system runs in real-time, but that it does so on limited hardware resources available on a test vehicle, enabling autonomous operation by using live sensor data. In order to meet the requirements for robustness and redundancy at algorithmic level, our perception system features parallel processing pipelines for camera and LiDAR data. We develop an independent solution for 3D object detection by processing LiDAR measurements. We also provide an enhanced perception solution, achieved by processing the spatio-temporal and appearance-based representation (STAR) obtained through low level fusion of the geometric data from LiDARs and semantic information obtained from RGB cameras.

**Figure 2:**
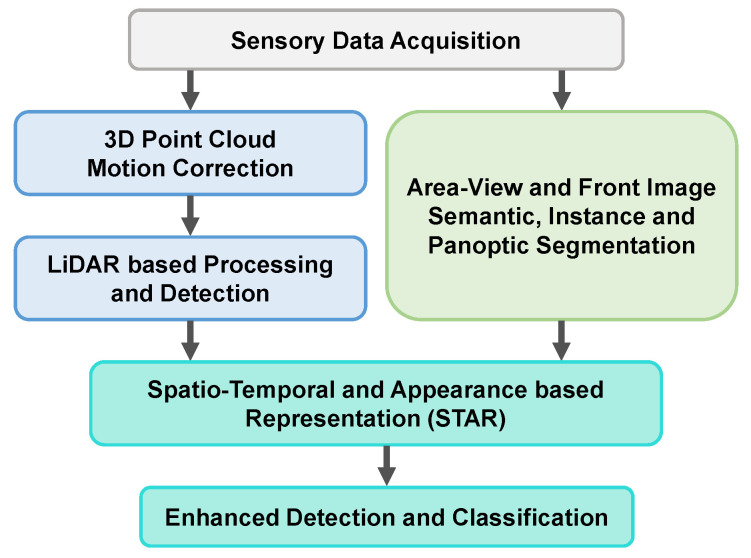
Overview of the enhanced perception system’s main components and their core interconnections: after data acquisition and pre-processing, 3D LiDAR data is processed independently of the 2D data up to the low-level fusion step which integrates the semantic information derived from the images with the corrected 3D measurements, yielding the STAR data representation which is further used for enhanced 3D detection and classification.

This perception solution builds upon our previous work focused on generating semantically enhanced point clouds [3], with large improvements to the capacity to extract semantic information from the scene, as well as an optimized implementation for real-time operation on limited hardware resources that handles all associated tasks: data pre-processing (data batch formation along with continuous monitoring of data availability, LiDAR motion correction, image undistortion), semantic information extraction, 3D point cloud segmentation, low-level fusion of 3D-point geometric data with 2D semantic information and, finally, the enhanced perception consisting of 3D object detection refinement and classification. We adopt a low-level fusion approach in order to retain a larger number of joint multi-modal features, which are typically lost in higher (e.g., object) level fusion schemes. As we aim to fuse raw 3D and 2D measurements as well as semantic information extracted from the camera images, we are constrained to performing the low-level fusion later in the processing chain, in order to maintain real-time capabilities on the imposed hardware resources. This allows processing the 3D and 2D modalities in parallel up to the fusion step. While an early-low-level fusion design could enable a more advanced segmentation of the enhanced point cloud, stalling the processing pipeline until the computationally intensive semantic segmentations are generated would dramatically increase the final latency of the results. In order to maintain real-time capabilities on the imposed hardware resources, we adopted this parallelized, late-low-level fusion architecture for the enhanced perception solution. The independently obtained 3D and 2D segmentations, together with the semantically enhanced point cloud, can be used in other parallel perception and higher level tasks. An overview of the enhanced perception system is presented in Figure 2.

For achieving the goals of the UP-Drive project, the Environment Perception System was designed based on the following specifications:

- Vehicle Operations
  1. Automated driving restricted to urban areas with 30 km/h speed limits imposed
  2. The driving areas have been previously mapped
  3. Limited hardware resources
  4. The software stack, including the enhanced perception, is running under ADTF (Automotive Data and Time-Triggered Framework [4])
- Expected Contributions in Perception
  1. 360° multi-modal and robust perception including detection and object classification
  2. Enhanced perception through low-level fusion of geometric and semantic data
  3. Independent processing of cameras and LiDARs
  4. Suitable frame rate (above 5 FPS) output to enable real-time operation

## 2. Related Work

This past decade proved to be one of rapid growth for the autonomous driving sector, in both the academic and industrial sectors. Companies such as Waymo (Google), Zoox and Tesla have made progress towards achieving functional commercial products, while independent research teams have also contributed greatly to the various advancements. This prompted the creation of multiple publicly available datasets (e.g., [5,6,7,8]) designed for aiding development of solutions for various tasks such as environment perception, object tracking and localization.

Recently, in [9], the authors conduct a comprehensive survey of available datasets, while providing a thorough analysis of the various deep learning based approaches for object detection and semantic segmentation. They compare different sensor modalities and the associated processing methods, further discussing the challenges of data fusion, such as the stage at which it is best to perform it. They recognize that low-level fusion schemes can achieve higher performance by better utilizing the raw information at the cost of lower model flexibility.

Some approaches forgo multi-modal perception, and hence the associated data fusion steps. Typically, these use high density LiDARs for gathering both 3D information as well as some texture information. In [10], the authors present a traditional solution based on a single high-density LiDAR, tailored for urban environments, that separates stationary features (represented in a multi-layer grid) from the dynamic objects which are subsequently tracked. The authors of [11] also argue for the utility of multi-layer grid maps when presenting a method for semantic segmentation of LiDAR scans using a convolutional neural network.

The majority of traditional (non-learning based) solutions for 3D obstacle hypothesis generation from LiDAR data rely on grouping based on vicinity criteria and fitting a L-shape model to the bird’s-eye view of LiDAR measurements for orientation detection. The problem of using multiple high-density LiDARs for perception is an open research subject and not dealt with frequently. In [12] the L-shape is fitted to the top view of the LiDAR measurements for generating obstacle proposals. In case of multiple LiDARs, the authors propose the back-projection of obstacle proposals to camera-image space for fusion. The back-projection technique can be less appropriate when the camera and LiDARs have significantly different mounting positions/viewpoints on the ego vehicle, and requires additional time for the projection and image space fusion. In [10] obstacle measurements from a single high density LiDAR are clustered using a polar grid with vertical compression (each cell can contain multiple occupied intervals). Voxel based representations are also proposed to represent and process LiDAR measurements, but mainly for static/dynamic discrimination and with single high-density LiDAR setups. In [13] the measurements from a single high-density LiDAR are converted to a voxel space that is used, in a temporal fusion approach, to discriminate between static and dynamic measurements. Removing dynamic obstacles from the LiDAR measurements (urban scenes with pedestrians) is also done in [14] by using a voxel representation combined with sphere quadtrees. An efficient voxel based fast segmentation algorithm is presented in [15] for detecting planar surfaces in scenes with very high density scans of buildings (or similar structures). The approach is, however, not meant for real-time processing as it takes tens of seconds on average per frame.

Dynamic grid based methods were also proposed. A particle-based solution was proposed in [16] for stereovision systems, by modeling a dynamic elevation map with a set of particles (each particle has as features the position, speed and height). In [17], a new dynamic grid approach was proposed, that relies on a Dempster–Shafer evidence framework, with multi-hypotheses evidential representation, persistent grid representation of the static environment, and a particle-tracking scheme that is applied only to dynamic areas. A random finite set (RFS) representation for the state of the dynamic grid cells is presented in [18]. Upon the RFS representation, a probability hypothesis density/multi-instance Bernoulli filter is proposed and implemented with particles.

Deep-learning-based object detection methods that use 3D LiDAR data may exhibit longer inference times or use more powerful hardware due to expensive 3D convolutions. Methods such as the single stage network PointPillars [19] achieve good detection and execution performance by encoding the 3D cloud into sparse pseudo-images that lend themselves to processing through 2D convolutions. In [20], a new deep learning end-to-end architecture is proposed, called Flownet3d, which is able to learn and detect the scene flow from consecutive frames. Camera-only solutions have the benefit of lower hardware costs, yet extracting the 3D information is more challenging. In [21] the authors propose a stereovision based Object Detection solution that extracts the 3D Bounding Boxes from an elevation grid map obtained by fusing disparity information with a CNN-generated semantically segmented image.

Multi-modal solutions that combine multiple data streams are still considered the best candidates for achieving autonomy, scoring high in accuracy and robustness. Moreover, adverse conditions impact sensing devices asymmetrically, a fact which can be exploited using methods such as in [22]. Here, the authors propose a deep-learning sensor-fusion architecture that performs well in difficult weather by using LiDAR, RADAR, RGB and gated cameras. Instead of detecting and characterizing the type of conditions, features are exchanged taking into account the entropy in each sensor’s measurement (heavily affected modalities exhibiting low entropy).

Another fused, CNN-based approach is presented in [23] where the authors extend LaserNet [24] to incorporate 2D image features extracted by an auxiliary network which are then mapped to 3D points. The enriched 3D points are then passed to LaserNet, obtaining a segmented scene and object detections with better performance than the LiDAR-only solution. In contrast, another category of fused solutions uses 2D detection networks to generate boxes that are then used to select the 3D points that project over them, which are subsequently used for extracting the object bounding box. Such a system is presented in [25] where the 3D detections are generated using candidate points through a RANSAC approach based on some generalized car models. The best proposal is then fed to another CNN that refines the box.

For the task of semantic 2D image segmentation, fully convolutional networks (FCNs), in which fully connected layers are replaced with convolutional layers, are widely used. The studies [26,27] capture context cues by including dilated convolutions in the last residual blocks and propose the Atrous Pyramid Pooling (ASP) with parallel dilated convolutions. Deformable convolutions [28] learn the dilation rate of convolutions instead of using a fixed rate. Spatial Pyramid Networks (PSPNet) [29] learn long-range and multi-scale information by applying parallel pooling operations. Although dilated FCNs obtain state-of-the-art results on public benchmarks such as Cityscapes [30], Mapillary Vistas [31] or COCO [32], they have a large memory footprint since the learned feature maps have a high output resolution. The encoder–decoder [33,34] architecture yields faster inference speed by employing a feature extractor network in the encoder and a lightweight decoder which learns to recover spatial resolution. Automated vehicles require real-time processing. Therefore, we integrate in our UP-Drive solution an optimized ERFNet [33] encoder–decoder network for semantic segmentation of the four unwarped area-view images.

Instance segmentation networks, which delineate each distinct object, can be classified into two-categories: proposal-based, which detect candidate regions of interest which are further segmented; and proposal-free, in which pixels belonging to the same semantic segment are clustered into instances based on the distance in an embedding space. Mask R-CNN [35] is the most representative proposal-based instance segmentation network, and achieves top performance on public benchmarks. The network extends the two-stage object detection network, Faster R-CNN [36], with a convolutional mask prediction head. Single-stage instance segmentation networks based on single-stage object detectors have simplified inference pipelines and employ specialized losses [37], or propose the use of prototype masks [38] in order to reach the performance of two-stage networks. Proposal-free networks are faster but have decreased accuracy. The subjects of [39,40] learn an embedding space by regressing pixel offsets to instance center and then a clustering step follows. Instance segmentation as a graph partition problem has been tackled in SSAP [41,42]. In [43,44] networks are proposed that learn to fit polygons around instances. Proposal-based methods show superior results, therefore we employ RetinaMask [37] for instance segmentation of front area-view image and front narrow FoV image. RetinaMask, the single-shot network, answers the requirements of our system in terms of accuracy and time and offers the advantage of simplified inference pipeline, which allows for further acceleration with deep learning inference engines [45].

The remainder of the paper is structured as follows: Section 3 presents in detail the proposed perception system, covering the hardware setup, software architecture and pre-processing functions, the 2D and 3D processing chains, the low-level fusion step for building the STAR representation and, finally, the classification and enhanced detection step. Section 4 contains the 2D and 3D evaluation procedures and the obtained results, along with timing information. Section 5 concludes the paper by outlining the main results and expected future improvements.

## 3. Proposed Approach for the Enhanced Perception System

### 3.1. Test Vehicle Sensory Setup

**Figure 3:**
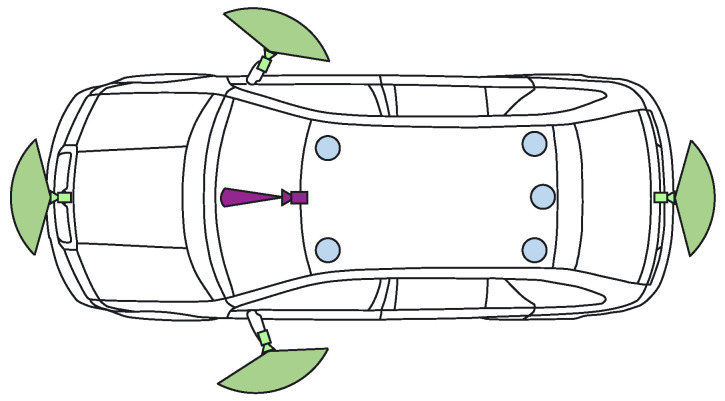
Sensor payload of test vehicle: 360${}^{\circ }$ LiDARs in blue, area-view system with fish-eye RGB cameras in green, narrow FoV camera in purple.

The demonstrator used in the UP-Drive project is based on a fully electric Volkswagen eGolf outfitted with industrial computers in the trunk space, which can take over all controls of the vehicle. The perception task is carried out on a small form factor PC that features a quad-core processor running at 3.60 GHz and a NVIDIA GTX1080 GPU. Figure 3 shows the sensor payload layout used by the enhanced perception solution: area-view system (also known as surround view, increasingly common on new vehicles) comprising of four cameras, one narrow field-of-view (FoV) camera and five LiDAR scanners. Ego-motion data is also available based on vehicle odometry (without GPS).

The four area-view (AV) cameras feature 180° horizontal FoV fish-eye lenses, enabling them to capture a surround view of the vehicle. The acquisition of 800 × 1280 px RGB images is externally synchronized between all AV cameras, with very low jitter. The additional narrow FoV camera is mounted in the position of the rear-view mirror and captures 1280 × 1920 px RGB images with a 60° FoV. It is also hardware synchronized with the AV system, complementing detection at longer ranges. Three-dimensional geometric information is captured by the five LiDAR scanners, three of which feature thirty-two vertical scan beams (asymmetrically spaced), while the middle-rear and right-rear scanners use sixteen beams.

Offline calibration [46,47] of the cameras and LiDARs provides intrinsic parameters of the cameras (radial distortion, focal length) and extrinsic geometric parameters (position and orientation in 3D) of cameras and LiDARs. Camera intrinsic calibrations are done using a standard checkerboard calibration pattern. The extrinsic calibration of the cameras provides the relative positions and orientations of the cameras with respect to a 360° LiDAR master sensor and is achieved with the aid of a set of markers mounted on the walls of a calibration room. The extrinsic 360° LiDAR calibration is also carried out in the calibration room and provides the relative position and orientations of each LiDAR with respect to a chosen master LiDAR (the front left sensor). Finally, the sensor coordinate system is registered with the respect to the car coordinate system.

### 3.2. Perception System Software Architecture

**Figure 4:**
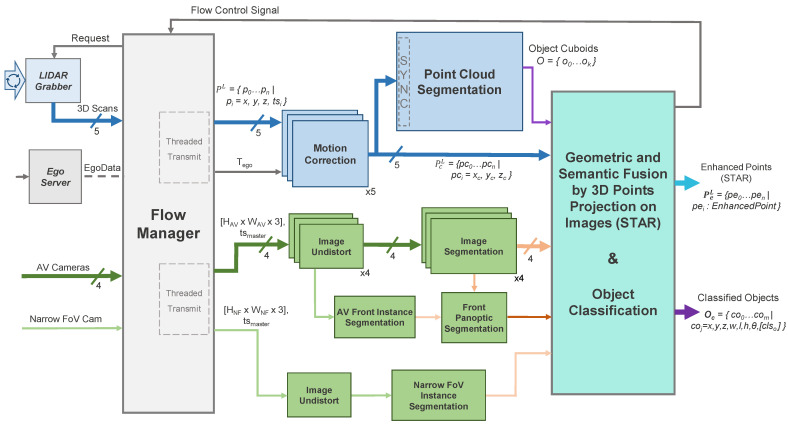
Data flow architecture of the proposed enhanced perception system: 3D geometric information (blue, top part) is processed in parallel with 2D camera images (green, bottom part) before the final merger of the two.

Figure 4 illustrates the enhanced perception system’s architecture, where the necessary operations are subdivided into a series of modular entities, all of which were implemented as plugins in the ADTF Automotive Framework [4] using the C++ programming language. The data inputs are aggregated by the Flow Manager which controls the two processing chains: the operations on the 3D point cloud (light blue) and those on the 2D images (light green).

The two sensor modalities are handled in parallel on limited hardware by delegating the 3D processing to the CPU and the camera processing to the GPU. The final plugin performs data fusion of the intermediary results, forming the basis for the final detection refinement and classification step. A set of classified objects $O_{c}$, containing both static and dynamic type detections is outputted, along with the semantically enhanced 3D point clouds (STAR) $P_{e}^{L}$, each corresponding to a LiDAR scanner *L*, which are further used in other modules of the system (e.g., curbstone detection [48] and localization [49]). Classified objects $co_{j}$ are represented as cuboids defined by their center (x,y,z), dimensions (w,l,h), orientation (θ) and ordered classification vector ($\left[cls_{o}\right]$) which features the most likely class predictions. The enhanced points use the EnhancedPoint data structure (detailed in Algorithm 1), which adds information both from the 2D domain as well as from the 3D segmentation (the object ID and class if the point is part of a segmented detection).
**Algorithm 1:** STAR 3D point enhancement representation. **struct {**(  **float** x,y,z;  **bool** isEnhanced;  **uInt8** r,g,b;  **uInt16** u,v; \\(projection coord.(  **uInt16** instanceID;  **uInt8** semClass;  **uInt16** objID;  **uInt8** objClass; **}**
*EnhancedPoint*;(

### 3.3. Pre-Processing Functions

The **Flow Manager** module performs multiple tasks: data and execution stream synchronization, as well as memory and thread management. We improve on our previous work in [50] based on continuous monitoring of the system running in real-time onboard the test vehicle. In order to reduce, as much as possible, the latency between the acquisition of raw measurements and the final results being outputted, we decided to forgo the use of point cloud buffers for each LiDAR scanner as these introduced additional delays due to the behavior of upstream components. Instead, we introduce a data request mechanism to communicate with the raw LiDAR data grabbers. Thus, a new data batch is formed by requesting the 360° point clouds from the LiDAR Grabber once processing completes for the current batch (signaled by the Flow Control Signal). The request is based on the batch’s master timestamp, corresponding to the (synchronized) acquisition timestamp of the most recently received set of images, $ts_{master}$. The response to the Flow Manager’s request consists of point clouds from every LiDAR *L*, where the last scanned point $p_{n}$ in each cloud was measured (roughly) at the master timestamp (consequently, the first scanned point will have been measured approximately 100ms earlier, as the scanners operate at a 10 Hz frequency).

Once all the required inputs are gathered, the Flow Manager plugin bundles the calibration parameters and ego motion data and sends them for processing on two independent execution threads, one for each sensor modality. The ego motion is provided as a 4 × 4 matrix $T_{ego}$, comprised of rotation and translation components that characterize the vehicle’s movement between the moment of image acquisition going back to the earliest scanned 3D point included in any of the five point clouds. The plugin also performs memory management and handles dropouts in sensor data: the system continues to function as long as the cameras and at least one LiDAR are online, while also being able to recover once offline sensors become available.

Camera images first undergo an **image undistortion** pre-processing step that produces undistorted and unwarped images that better represent the scene, based on the camera’s projection model, as detailed in [3]. While we have experimented with single- and multi-plane representations of the virtual undistorted imager surface, the best results were obtained when considering a cylindrical representation in which the axis is aligned with the ground surface normal, defined by the equations in Equation (1) (here, α represents the horizontal field of view of the camera, with β=αH/W). Using look-up tables, this step is performed in 1 ms for each camera.

(1) $$\begin{array}{cc} X(u,v) & =sin(-\alpha +\alpha \;\cdot \;u/(W-1))\\ Y(u,v) & =-\beta +\beta \;\cdot \;v/(H-1)\\ Z(u,v) & =cos(-\alpha +\alpha \;\cdot \;u/(W-1)) \end{array}$$

For 3D data, **motion correction** is the process of temporally aligning the sequentially-scanned points $p_{i}$ to the master timestamp of the data batch (Equation (2)), thus eliminating the warping induced by ego vehicle movement during the scan (obtaining $pc_{i}$). Besides correcting geometric distortions, this step also enables a pseudo-synchronization between the two sensing modalities, temporally aligning the point clouds to the moment of image acquisition (represented by the master timestamp)—the unknown movements of dynamic objects in the scene limiting the possibility for full synchronization.

(2) $$pc_{i}=C_{i}\;\cdot \;p_{i}$$

The procedure carried out by the correction plugins (one for each LiDAR *L*) is a computationally-optimized version of the one we presented in [3], completing the task in approximately 2.5 ms, yielding corrected point clouds $P_{c}^{L}$: the correction transform $C_{i}$ is computed using the matrix exponential/logarithm functions [51] applied on the ego-motion transform $T_{ego}$, based on the time difference $\Delta_{i}$ between master and point $ts_{i}$ timestamps (Equations (3) and (4)). To reduce the processing time of each motion correction plugin, we use look-up tables to compute approximations of $C_{i}$, each of which can be applied to small groups of 3D points captured in very rapid succession one after the other, without decreases in quality. We also employ the OpenMP library to perform computations in parallel. This does require a fine tuning of the maximum number of workers allowed to be used by each plugin instance, based on the system load of the host computer. Each worker is assigned a subset of points from the cloud, in order to minimize computational overhead.

(3) $$\Delta_{i}=ts_{master}-t_{i}$$

(4) $$C_{i}={\left(T_{ego}\right)}^{-\frac{\Delta_{i}}{\Delta_{0}}}=exp\left(-\frac{\Delta_{i}}{\Delta_{0}}\;\cdot \;log\left(T_{ego}\right)\right)$$

### 3.4. 2D Panoptic Segmentation

We develop a robust, fast and accurate 360${}^{\circ }$ deep-learning-based 2D perception solution by processing images from the five cameras mounted on the UP-Drive vehicle. Our 2D perception module’s goal is to detect static infrastructure such as road, sidewalk, curbs, lane markings, and buildings, but also to detect traffic participants. This is achieved with image segmentation which extracts semantic and instance information from images. We also solve inconsistencies between semantic and instance segmentation and provide a unified output in the form of the panoptic segmentation image. In the case of wide-angle area-view cameras that provide the 360${}^{\circ }$ surround view, a large extent of the scene is captured, but the apparent sizes of objects are smaller compared to narrow FoV cameras. Thus, the detection range of segmentation algorithms of area-view images is limited to the near range around the vehicle. In use cases such as parking or navigation at very low speed, area-view images provide the necessary range. However, detecting distant objects is important when driving at higher speed in urban environment. Therefore, we also utilize the narrow 60${}^{\circ }$ FoV frontal camera, mounted behind the windshield, in order to extend the detection range. A detailed description of our 2D perception solution is presented in [52].

Our hardware resources are limited and our system requirements impose that our 2D perception solution runs in at least 10 FPS. In order to satisfy these constraints, we develop semantic image segmentation for the area-view images and instance segmentation only for the front area-view image and the front narrow FoV image. Panoptic image segmentation is implemented for the front area-view image. The instance and panoptic segmentation can be easily extended to other views when using more powerful hardware.

**Figure 5:**
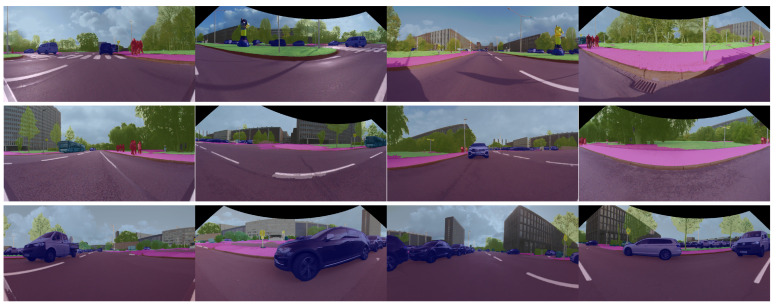
Semantic segmentation of the four area-view images: front, right, rear and left. The semantic perception provides 360${}^{\circ }$ coverage, where each image has a 160${}^{\circ }$ FoV.

For semantic segmentation we implement a lightweight fully convolutional neural network (FCN) based on ERFNet [33], which has an encoder–decoder architecture. Results of the semantic segmentation for the four cameras are illustrated in Figure 5. For instance segmentation we employ the single-stage RetinaMask [37] network.

**Figure 6:**
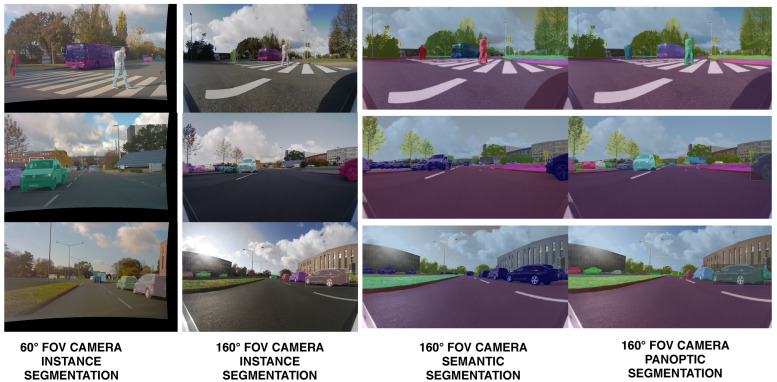
Comparison of the front narrow FoV and the front area-view instance segmentation. The apparent sizes of objects is larger in the 60${}^{\circ }$ FoV image than the area-view image. By introducing the narrow FoV image, we increase the instance segmentation depth range. We provide instance, semantic and panoptic segmentation on the front area-view image.

In order to solve conflicts between the semantic class provided by the instance and semantic segmentation, we develop a fusion scheme [53] for panoptic segmentation. We observe the following problems that are solved by our solution: low-resolution instance masks (28×28) are upsampled to match the input image resolution and results in raw object borders, semantic segmentation of large objects is problematic and classes belonging to the same category are often confused. The idea is to match pixels from the semantic and instance segmentation at category level and perform a region-growing algorithm in order to propagate the instance identifier and instance segmentation class, which is more stable. Figure 6 shows the output of the instance segmentation process performed for the two cameras, as well as the panoptic segmentation resulting from the fusion with the semantic segmentation.

### 3.5. 3D Point Cloud Segmentation

We adopt a two-step strategy for 3D point cloud segmentation: first, the road surface information is extracted from each LiDAR cloud individually, which provides a first separation between LiDAR measurements originating from the road and those from obstacles. This is then followed by a 3D obstacle detection step for which we propose a voxel representation that supports fusion across LiDAR sensors. Thus, we exploit the different viewpoints of each LiDAR scanner in order to obtain a better detection of obstacles in a single pass. Non-road LiDAR points obtained in the first step are fused (and densified) into a single voxel space representation. For each 3D obstacle we provide both the traditional cuboidal representation as well as blobs of connected voxels, which can more accurately capture the occupied space of hanging or non-cuboidal obstacles.

**Figure 7:**
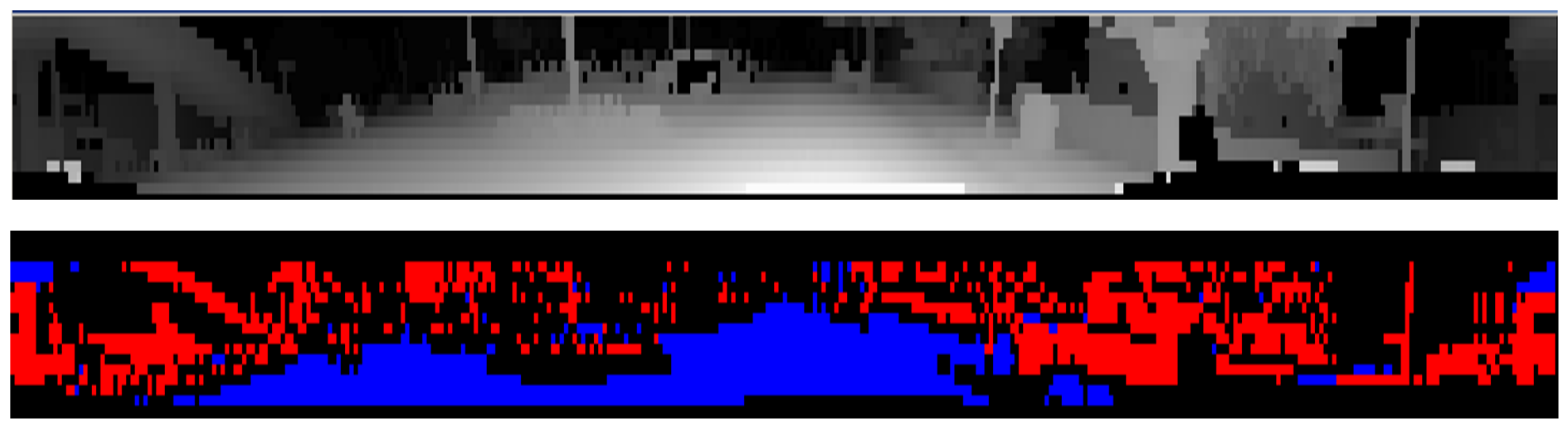
(**Top**): Layer/channel representation of the front left LiDAR output (the logarithm value of the inverse depth is shown for visualization), (**Bottom**): Preliminary classification results (road—blue/obstacles—red).

Figure 7 top, illustrates the initial layer/channel representation of LiDAR data on which the road surface detection is carried out, where 3D measurements are ordered by their layer (vertical) and channel (horizontal) angles. Figure 7 bottom, shows the preliminary road/obstacle classification of the panoramic data which takes into account the local slope information.

**Figure 8:**
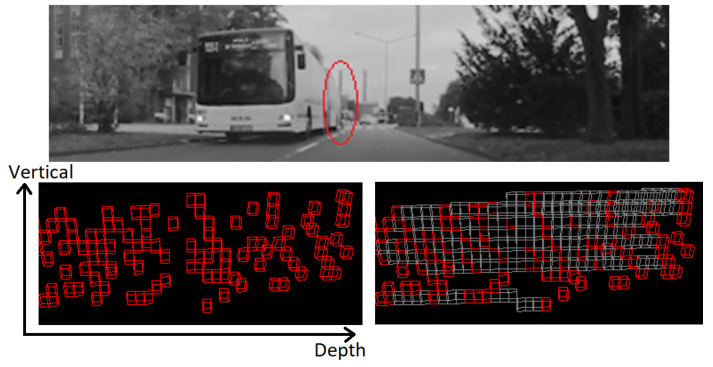
The farthest part (encircled with red, (**top image**)) of the lateral side of the bus has consecutive LiDAR measurements that are not connected in the voxel space ((**bottom-left image**): side view, red voxels). After densification, the introduction of the intermediate voxels (gray) provides an increased level of connectivity ((**bottom-right image**): side view).

The voxel representation covers a space of interest centered on the ego vehicle, with a square shape in the bird’s-eye view. The top view area covered is 160 × 160 m, with a voxel size of 16 × 16 × 16 cm, with the ego vehicle in the center. Each obstacle measurement is inserted into the voxel space, and voxels are marked as obstacles accordingly. However, one important issue must be accounted for: depending on the orientation of an obstacle facet relative to the LiDAR, or the distance from the ego car, obstacle measurements that are adjacent in the layer/channel space might not be connected in the Euclidean voxel space (Figure 8).

The densification of the voxel space is achieved by inserting intermediate obstacle voxels between the originally-adjacent LiDAR measurements. Both vertical and horizontal connectivity are exploited. Two obstacle measurements are adjacent in the following configuration:

(a) (vertical) Adjacent layers and the same channel, and the 3D distance between the measurements is below a threshold
(b) (horizontal) The same layer and adjacent channel values, and the 3D distance between the measurements is below a threshold.

For the second situation (horizontal connectivity), an additional constraint must be imposed regarding the planarity of the surface where the intermediate voxels are inserted. This is needed in order to avoid unwanted joining of nearby independent obstacles. The local angle of the 3D measurements is evaluated along each layer (from the previous, current and next channel measurements). Intermediate voxels are inserted only for those obstacle measurements where this angle is close to 180 degrees (should belong to a surface that is close to planar). Computing the location of intermediate voxels that must be inserted can be quickly solved using Bresenham’s line algorithm (3D version).

**Figure 9:**
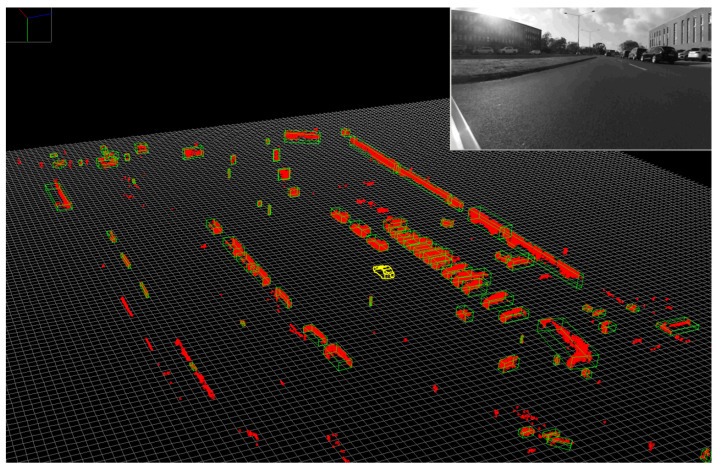
Individual obstacles are detected in the voxel space (red voxels), and valid obstacles are represented as oriented cuboids (green).

By densifying the voxel space, we ensure that measurements adjacent in the panoramic representation are connected, by having intermediate voxels forming uninterrupted paths between them even in cases with no intermediate measurements. The intermediate and occupied voxels allow the extraction of 3D obstacles as connected components. These are found through a breadth-first search which generates a set of candidates for which multiple features (size, voxel count, LiDAR 3D point count, etc.) are calculated, which allows for some of them to be discarded if their features are outside of expected ranges. Finally, for each obstacle blob, the oriented cuboid is computed with a low complexity approach based on random sample consensus fitting of L-shapes, described in [54]. Results are shown in Figure 9.

### 3.6. Low-Level Fusion: Spatio-Temporal and Appearance Based Representation

**Figure 10:**
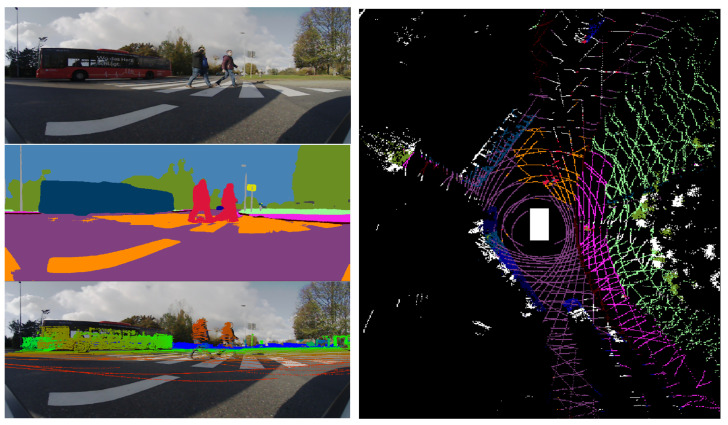
The Low-level fusion process: Raw and semantic information from the camera is fused with the projected 3D points (left column, from (**top**) to (**bottom**)) to produce an enhanced 3D point cloud (STAR, (**right**)).

The **Geometric and Semantic Fusion** module aggregates data from the two execution threads and generates the spatio-temporal and appearance-based representation (STAR) by projecting all the motion-corrected 3D points onto every raw and segmented image using the associated calibration parameters of each sensor (see Figure 10). This process, which attaches RGB and semantic information to all points that project onto the 2D data, can become a computational bottleneck, prompting us to use a complex, multi-threaded execution scheme, that takes place for each received 3D cloud once 2D segmentation results become available.

Equations (5)–(7) present the mechanism for projecting 3D points $pc_{i}$ onto the unwarped images based on the cylindrical image plane representation, using the camera intrinsic matrix $K_{cam}$ and the 3D coordinate transform $T_{cam}^{L}$, provided by the calibration procedure for each pair of camera cam and LiDAR *L*. Here, the function point_to_cylinder computes coordinates at which rays extending towards $pc_{i}$ intersect the cylindrical surface of the (modelled) image plane.

(5) $${\left[u,v,1\right]}^{T}=K_{cam}\;\cdot \;point\_to\_cylinder\left(T_{cam}^{L}pc_{i}\right)$$

(6) $$point\_to\_cylinder\left({\left[x_{c},y_{c},z_{c},1\right]}^{T}\right)={\left[asin\left(x_{c}/r\right),y_{c}/r,1\right]}^{T}$$

(7) $$r=\sqrt{x_{c}^{2}+z_{c}^{2}}$$

**Occlusion handling** is an important practical problem which arises during sensor fusion and we address it with two different approaches. They both use semantic and depth information: one fast approach relying on projecting onto a low-resolution virtual image and one more costly approach which applies corrections to each image column.

Semantic labels from occluding objects seen in camera images can be mistakenly propagated to 3D points belonging to the occluded objects during the information fusion process between the LiDAR point cloud and semantic segmentation images. The occlusion handling algorithm presumes objects to be either static or that their position has been corrected (which in turn requires additional information).

The **sparse depth map** (raw) representation, denoted by D(x;y) is obtained by projecting the 3D measurements onto each image, sparsely adding distance information to pixels where possible, with the other pixels marked accordingly. An intermediary operation aims to increase the density of the depth map by considering cells of fixed size *s* (e.g., 10 × 10 px) and constructing a lower-resolution depth map by taking the minimum distance in each cell. In practice, this can be implemented by creating the low-resolution image directly, which reduces computation time significantly and achieves the same results. The low-resolution image has values equal to the local minimum in an *s* by *s* box:

(8) $$D_{lr}\left(x;y\right)=min_{i;j}D(i;j)\;|\;x=[i/s];y=[j/s]$$

Besides sparsity, another common problem is the lack of measurements due to non-reflective surfaces of objects from the scene. To address this issue we propose two correction schemes: one based on the dilation of the measurements and the other based on convex hull estimation.

Since LiDAR point layers fall farther apart onto objects which are closer, measurements are dilated in the horizontal and vertical direction based on the distance. The dimensions of the dilation kernel are given by: $d_{rows}=min\left(4;20/dist\right),d_{cols}=min\left(1;5/dist\right)$, where dist is the distance of the 3D point, making the the window size inversely proportional to it (the scalar values were selected empirically, with a higher kernel size needed on the vertical direction due to higher sparsity). This operation does not affect the execution time significantly since it only applies to points within a 20 m range.

Semantic information allows us to treat each object class differently. Occluding objects can only be from the following semantic classes: construction, pole, traffic light, traffic sign, person, rider, car, truck, bus, train, motorcycle, bicycle, building or vegetation. Point measurements from other classes, such as road or lane markings, cannot occlude other objects.

**Figure 11:**
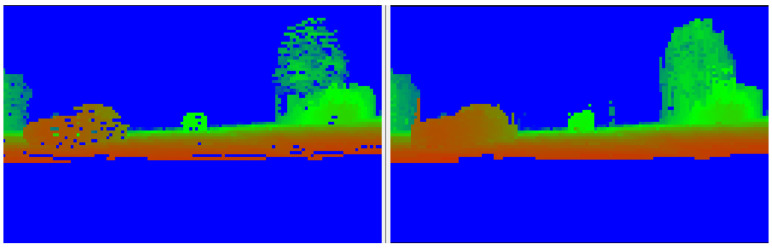
Low resolution depth map and its corrected version - scene depicts a car close by on the left, a car in the middle farther away and foliage on the right. Color encodes the distance from red (close) to blue (far), up to 20 m. Positions with no measurements default to infinity.

Occlusions are detected using the generated dense depth map by simply marking projected points which have a larger distance than the corresponding cell from the depth map as occluded. Semantic classes associated to such points should be ignored as they originate from an occluding object. Figure 11 illustrates the resulting low-resolution depth map.

An alternative, more computationally costly, approach was also implemented. It performs a column-wise completion and relies on estimating the lower envelope of the measurements for a single object based on the semantic labels. More precisely, we consider each column from the depth map and segment it according to the semantic labels. For each segment, after finding the lower envelope we update the depth values to match those from the envelope. This predicts the depth linearly between two measurement points and eliminates farther measurements.

### 3.7. STAR-Based Classification and Enhanced Detection

Once the LiDAR measurements are enhanced with semantic information while also considering occlusions, the process of **object classification** can be performed in order to provide class labels to each object cuboid. In order to increase robustness to outliers caused by mislabeled 3D measurements (such as those on the border of dynamic obstacles or on thin objects), a statistical method is adopted. The following steps are performed for computing the semantic class for each detected object:

- The semantic class is first transferred from the individual 3D measurements to their corresponding voxels, handling conflicting measurements by labeling the entire containing voxel as unknown
- A semantic class frequency histogram is generated for each 3D obstacle, based on the contained voxels, enabling a statistical based approach for the final decision step:
  - The most frequent class is selected as the 3D object class
  - Up to 3 additional, most likely classes can be also provided, allowing further refinement of the object class during subsequent tracking processes

**Figure 12:**
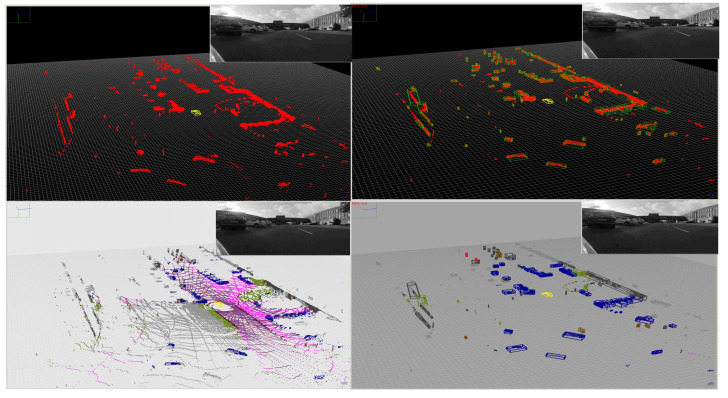
Results of Object Detection and Classification. **Top-Left**: Input LiDAR Point Cloud, **Bottom-Left**: STAR Point Cloud, **Top-Right**: Segmented Objects, **Bottom-Right**: Classified Objects.

Results for obstacle classification are presented in Figure 12. The enhanced semantic information of the 3D points can be further exploited to refine the detected objects by separating grouped obstacles of different classes or different instances of the same class. A strategy was developed to analyze the distribution of classes or instances in an obstacle. If at least two dominant classes/instances are detected (frequency over a threshold), then new centroids of individual obstacles are computed and voxels are reassigned to each obstacle (nearest centroid).

## 4. Evaluation and Results

### 4.1. 2D Evaluation

*Image Segmentation Dataset*: The image segmentation networks are trained and evaluated on the UP-Drive dataset. The dataset is large and diverse, and images have been recorded with our prototype vehicle in several cities, highways and country roads in northern Germany. Sequences were acquired during daytime, at various times of the day to capture diverse lighting conditions and during several months in three seasons: spring, summer and autumn. Moreover, recordings were taken in sunny and cloudy weather, but also in adverse weather conditions with heavy rain. The area-view dataset contains 19,562 frames from all four views which are manually annotated for semantic and instance segmentation. There are 23 semantic classes and 6 classes for instance segmentation. We create a training split with 15,782 images and a validation split with 3780 images. The narrow FoV front dataset is smaller and has been created in the last stages of the project. There are 1869 images which are annotated using the same methodology. We train on 1495 images and validate on 374.

*Evaluation Metrics*: For semantic segmentation, we employ the mean Intersection over Union (mIoU) metric. We evaluate instance segmentation using AP@[.5:.05:.95] (Average Precision over classes and 10 IoU levels).

*Inference Time*: We measure the inference time including all post-processing steps (such as NMS) on a NVIDIA GTX 1080 GPU with a batch of one image.

#### 4.1.1. Semantic Segmentation Results

The segmentation network is fully convolutional and can be easily optimized with the TensorRT library [45]. We also quantize the network in INT8, reducing the inference time four times at full image resolution. The results and inference times for semantic segmentation of area-view images are presented in Table 1.

**Figure 13:**
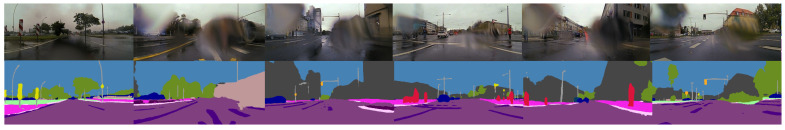
Semantic segmentation results in adverse weather conditions with heavy rain.

In Figure 13 we present qualitative results for semantic segmentation in adverse weather conditions with heavy rain. Despite the fact that raindrops partially cover the camera lens, we obtain good semantic segmentation results.

#### 4.1.2. Instance Segmentation Results

The limited hardware and the time constraints enable us to segment instances only from the front view, which provides the most useful information for navigation. In Table 2, we report the evaluation results of the instance segmentation network on the front area-view images. We train with multiple resolutions and obtain 36.8 box mAP, 30 mask mAP in 66 ms inference time. Although lowering the resolution decreases significantly the processing time, the accuracy is substantially degraded. Since the apparent size of objects in the area-view image is small, even for medium distance, we observe a decrease in detection range for small resolutions. Table 2 also includes the results for the narrow FoV camera instance segmentation network. In the final solution, we adopt the 832×416 image resolution and obtain 28.7% box mAP, 21.4% mask mAP with an inference time of 44 ms. The network is optimized with the TensorRT library in FP32 precision.

#### 4.1.3. Inference Time

We measure the inference time of the 2D semantic perception system in Table 3. Image unwarping of the four area-view images, along with the image undistortion, are implemented on the GPU and account for 6 ms processing time. The semantic image segmentation of the four area-view images takes 36 ms, the instance segmentation of the front area-view image takes 66 ms, while the instance segmentation of the front narrow FoV image takes 44 ms. Panoptic segmentation is fast and runs in 5 ms. Once the image segmentation for any image is available, we directly associate the semantic information with the 3D point cloud. The computational complexities for the modules are: 96 GFLOPs per image for semantic segmentation (before INT8 optimization), 380 GFLOPs for the front area-view instance segmentation and 162 GFLOPs for the front narrow FoV instance segmentation. A 10 FPS 2D perception system can be obtained with 360${}^{\circ }$ semantic segmentation of area-view images and instance segmentation of the front narrow FoV image.

### 4.2. 3D Evaluation

*Evaluation Data*: We perform the 3D object evaluation on a custom dataset. While evaluation on a public dataset would be ideal, the particular sensor configuration to which our perception pipeline is fine tuned to, both the Image and LiDAR chains, does not allow for this. Thus, we carry out the evaluation on a set collected by one of the vehicle demonstrators outfitted with the complete sensor payload. Objects of interest (i.e., cars, pedestrians, trucks, bicyclists) are manually annotated as cuboids in 3D space for comparison with the system’s classified output. Scenes were selected so that they best represented urban scenarios, with vehicle and pedestrian traffic, most of them recorded in damp conditions.

*Constraints*: Objects only partially visible in the LiDAR data were annotated based on a set of typical dimensions, even though the measurements covered only a portion of the entire cuboid. This decision stems from requirements of other intended uses of the annotated dataset. Another decision in performing the evaluation was limiting it to the road environment including the drivable and sidewalk areas in each frame, where the annotated objects can be found.

*Evaluation Metrics*: The traditional Intersection-over-Union metric, based on the intersection and union of areas (2D) or volumes (3D) between the detection and ground truth boxes, cannot accurately measure the agreement between the two due to the annotation strategy. To solve this, we propose a metric that is more resilient: a score is computed by counting the number of common (intersecting) 3D points of the cuboids being compared and dividing it to the number of total distinct 3D points that are inside any of the two volumes. Going further, we denote this metric as Point Intersection-over-Union (PIoU). While this measure is still sensitive to, for example, the number of ground points included in the cuboids, it is currently the best way to measure the localization capabilities. The PIoU metric enables evaluation of average, per frame, precision and recall, which then allow the computation of the average precision (AP) score.

*Results*: Table 4 shows the average, per frame, precision and recall values, with the perception area split into three intervals, at 25 and 50 m. When computing these scores, we considered a PIoU of 0.5 for objects closer than 25m and 0.3 for the rest. The classification and detection refinement shows a slightly lower accuracy and recall in comparison with the independent LiDAR based object detection, due to the added complexities of the classification task.

We evaluated the Average Precision (AP) metric by computing the area under the Precision-Recall curve, obtained by varying the threshold detections are considered valid, based on their associated confidence score. The obtained result of 71.65% covers the entire road area, up to a distance of 70m and considers all classes for the 1557 evaluated objects.

**Figure 14:**
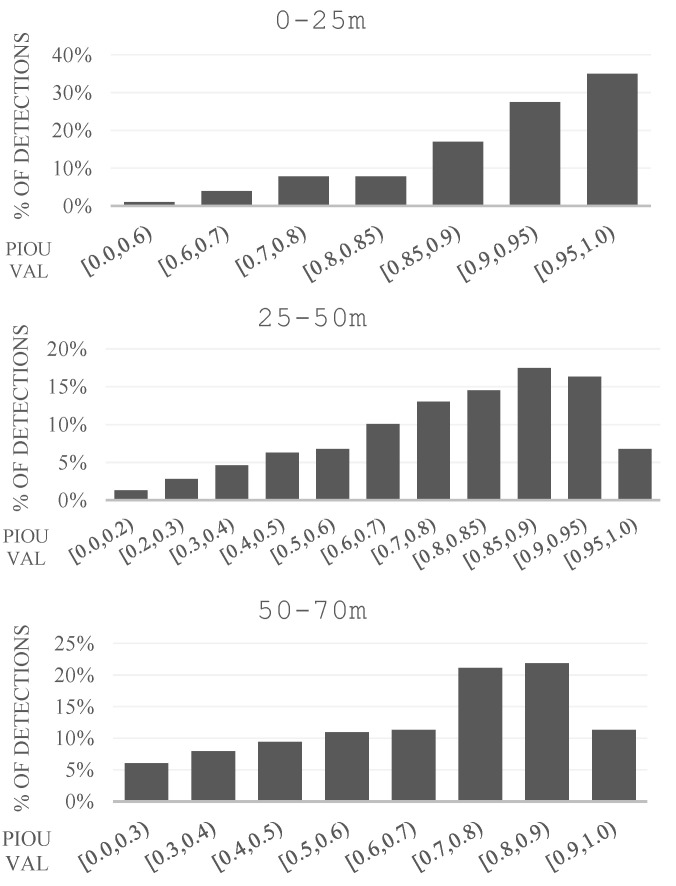
The Point IoU Histograms, for the three distance intervals.

Figure 14 shows how the PIoU score varies for detections at different ranges (computed for each detection’s best paired ground truth object, if one exists). The horizontal axis maps several ranges into which the PIoU value can fall, with the y-axis representing the percentage of detections out of the total number associated with each interval. For the close range, over 75% of detections have an associated PIoU value greater than 0.85, while the medium range interval exhibits over 68% of detections with a localization score of over 0.7, which drops to 53% for the longest range interval. At this range, 23% of detections are scoring below 0.5. When analyzing this data, it is important to note the fact that many of the evaluation frames were taken in wet conditions, where moving vehicles are trailed by a spray of water particles which are picked up by the LiDAR scanners. Our annotations exclude these trails, yet some detections include them, due to significant 3D point clusters present.

**Figure 15:**
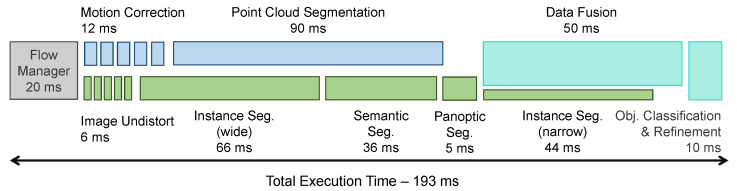
Execution flow and timing diagram with average processing times: the top blue 3D processing chain running on the CPU in parallel with the one for 2D data, running on the GPU.

Our software, running on its dedicated PC, outputted results at a rate of 5 FPS, or 6 FPS if the instance segmentation of the front wide angle camera was disabled (normal semantic segmentation still being computed). Figure 15 presents the average processing times for the individual components and the way the CPU and GPU threads are parallelized. It must be noted that this includes the data pre-processing step performed by the Flow Manager component (20 ms), which is necessary for online operation but is typically overlooked in offline-only solutions. Given the design constraints (areas with speeds limited to 30 km/h), coupled with the range of the perception system and the parallel operation over multiple processing nodes of the entire autonomous system’s components (perception, tracking, localization, scene understanding, navigation), the overall system latency was sufficiently low to allow proper operation in safe conditions.

The enhanced perception system was extensively tested on-board the test vehicle during drives in urban environments, with various adjustments being continuously added so that the full-stack of software which enabled autonomous driving performed in safe parameters. Unlike offline, sandboxed operations, giving the system control of the car meant taking extensive precautions for softly handling errors, enabling a graceful degradation in case any of the complex components failed. The fine-tuned software was operated for sessions longer than 60 min, with fewer than five interventions from the safety driver.

## 5. Conclusions

In this paper we have presented our multi-modal, multi-sensor, real-time-capable 360${}^{\circ }$ environment perception solution which has been successfully integrated onto the autonomous demonstrator developed in the UP-Drive project. The enhanced object detection and classification relies on the enhanced 3D point cloud representation (STAR). This, in turn, is obtained through the low-level fusion approach which combines geometric data from five LiDAR scanners with semantic, instance and color information obtained from a system of four fish-eye cameras covering the entire proximity of the vehicle, coupled with a narrow FoV front camera used for longer ranges. The two sensor modalities are processed independently, in parallel on the CPU and GPU, until the last fusion step, with the results being outputted at 5 FPS when running on the on-board hardware. This was enabled by a set of optimized deep learning based 2D semantic, instance and panoptic segmentation algorithms along with a 3D point cloud segmentation based on computer vision providing a rough object detection and classification. In the final step, the object detection and classification is refined through the use of the enhanced point cloud. By achieving these results, we fulfilled the overarching goals for the perception system in the project, thus enabling autonomous operation in dynamic urban environments.

The developed segmentation models, along with various optimizations of our system’s architecture, enabled the use of the entire Area View image area at a 1280×640 resolution with processing times of under 10 ms per image. The high-resolution increases the depth range and quality of the segmentation, as does the inclusion of the front-facing narrow-field-of-view camera. Our improvements regarding the execution times allowed integrating the instance and panoptic segmentation for the front-area-view and narrow-field-of-view cameras, contributing to a better and deeper semantic view of the scene. Future expansions in processing power will allow adding narrow-field-of-view cameras for all directions, as well as permit the use of more complex and more accurate models. Advances in automatic annotation generation, semi-supervised and un-supervised learning approaches will also improve the quality, consistency and diversity of the training sets.

In order to reduce processing time of the perception system, the 3D point cloud segmentation is done in parallel with the semantic segmentation. The enhancement of 3D points with semantic information allows for 3D detection refinement and accurate 3D object classification. More powerful hardware along with novel, faster 2D and 3D segmentation solutions could allow a serial approach starting with image semantic segmentation, followed by the fusion of 2D semantic information with the 3D geometric data and the 3D object detection, and classification carried out on the semantically-enhanced 3D point cloud. Improvements in the quality of sensor calibrations and synchronizations, in conjunction with optimizations to occlusion handling for dynamic objects, are to be developed further. Improving the robustness and accuracy of the enhanced perception solution can be achieved by increasing the variety and redundancy of the 3D and 2D sensors. This can be done by incorporating additional 3D sensors such as RADARs and solid state LiDARs, as well as multiple 2D narrow-field-of-view and far-infrared cameras.

## Figures and Tables

**Table 1:** Evaluation of the semantic segmentation network on area-view images corresponding to front, left, back, right views. 1280×640—INT8 (in bold) is integrated in the final solution.

Resolution	mIoU	Inference Time (ms)
1280 × 640—FP32	67.87	20
**1280 × 640—INT8**	**65.10**	**9**

**Table 2:** Evaluation of the instance segmentation network on area-view images corresponding to front, left, back, right views and narrow FoV images on the front view. Inference time is measured on a single image. The bolded configuration is integrated in the final solution.

Resolution	mAP Box	mAP Mask	Inference Time (ms)
Area-view images			
**1280 × 640—FP32**	**36.8**	**30**	**66**
832 × 416—FP32	30.1	24.8	44
640 × 320—FP32	26.4	21.6	33
Narrow FoV images			
960 × 604—FP32	30.2	22.8	58
**832 × 416—FP32**	**28.7**	**21.4**	**44**
640 × 320—FP32	23.4	18.3	33

**Table 3:** Time evaluation of the 2D semantic perception system.

Module	Inference Time (ms)
Image unwarping and undistort × 5	6
Semantic segmentation × 4	36
Instance segmentation front 160${}^{\circ }$ FoV image	66 (380 GFLOPs)
Panoptic segmentation front 160${}^{\circ }$ FoV image	5
Instance segmentation front 60${}^{\circ }$ FoV image	44 (162 GFLOPs)
Total	157

**Table 4:** Average Frame Precision and Recall results (%) at three different ranges, with and without taking into account the classification.

	Avg. Frame Precision			Avg. Frame Recall		
**Range (m)**	**0–25**	**25–50**	**50–70**	**0–25**	**25–50**	**50–70**
Detection & Classification	91.12	82.53	75.09	84.96	84.39	66.55
Detection	92.76	83.56	75.99	86.71	86.11	70.58

## Data Availability

Not applicable.

## References

[1] UP-Drive H2020 European Union Program—Grant 688652. https://up-drive.ethz.ch/.

[2] Buerki M., Schaupp L., Dymczyk M., Dube R., Cadena C., Siegwart R., Nieto J. Map Management for Efficient Long-Term Visual Localization in Outdoor Environments. Proceedings of the IEEE Intelligent Vehicles Symposium (IV).

[3] Varga R., Costea A., Florea H., Giosan I., Nedevschi S. (2017). Super-sensor for 360-degree environment perception: Point cloud segmentation using image features. Proceedings of the 2017 IEEE 20th International Conference on Intelligent Transportation Systems (ITSC).

[4] EB Assist ADTF-Automotive Data and Time-Triggered Framework. https://www.elektrobit.com/products/automated-driving/eb-assist/adtf/.

[5] Geiger A., Lenz P., Urtasun R. (2012). Are we ready for autonomous driving? the kitti vision benchmark suite. Proceedings of the 2012 IEEE Conference on Computer Vision and Pattern Recognition.

[6] Sun P., Kretzschmar H., Dotiwalla X., Chouard A., Patnaik V., Tsui P., Guo J., Zhou Y., Chai Y., Caine B. Scalability in perception for autonomous driving: Waymo open dataset. Proceedings of the IEEE/CVF Conference on Computer Vision and Pattern Recognition.

[7] Caesar H., Bankiti V., Lang A.H., Vora S., Liong V.E., Xu Q., Krishnan A., Pan Y., Baldan G., Beijbom O. nuscenes: A multimodal dataset for autonomous driving. Proceedings of the IEEE/CVF Conference on Computer Vision and Pattern Recognition.

[8] Huang X., Cheng X., Geng Q., Cao B., Zhou D., Wang P., Lin Y., Yang R. The apolloscape dataset for autonomous driving. Proceedings of the IEEE Conference on Computer Vision and Pattern Recognition Workshops.

[9] Feng D., Haase-Schütz C., Rosenbaum L., Hertlein H., Glaeser C., Timm F., Wiesbeck W., Dietmayer K. (2020). Deep multi-modal object detection and semantic segmentation for autonomous driving: Datasets, methods, and challenges. IEEE Trans. Intell. Transp. Syst..

[10] Rieken J., Maurer M. (2020). A LiDAR-based real-time capable 3D Perception System for Automated Driving in Urban Domains. arXiv.

[11] Bieder F., Wirges S., Janosovits J., Richter S., Wang Z., Stiller C. (2020). Exploiting Multi-Layer Grid Maps for Surround-View Semantic Segmentation of Sparse LiDAR Data. arXiv.

[12] Rangesh A., Trivedi M.M. (2019). No blind spots: Full-surround multi-object tracking for autonomous vehicles using cameras and lidars. IEEE Trans. Intell. Veh..

[13] Asvadi A., Premebida C., Peixoto P., Nunes U. (2016). 3D Lidar-based static and moving obstacle detection in driving environments: An approach based on voxels and multi-region ground planes. Robot. Auton. Syst..

[14] Schauer J., Nüchter A. (2018). The Peopleremover—Removing Dynamic Objects From 3-D Point Cloud Data by Traversing a Voxel Occupancy Grid. IEEE Robot. Autom. Lett..

[15] Huang M., Wei P., Liu X. (2019). An Efficient Encoding Voxel-Based Segmentation (EVBS) Algorithm Based on Fast Adjacent Voxel Search for Point Cloud Plane Segmentation. Remote Sens..

[16] Danescu R., Nedevschi S. (2013). A particle-based solution for modeling and tracking dynamic digital elevation maps. IEEE Trans. Intell. Transp. Syst..

[17] Steyer S., Tanzmeister G., Wollherr D. (2018). Grid-based environment estimation using evidential mapping and particle tracking. IEEE Trans. Intell. Veh..

[18] Nuss D., Reuter S., Thom M., Yuan T., Krehl G., Maile M., Gern A., Dietmayer K. (2018). A random finite set approach for dynamic occupancy grid maps with real-time application. Int. J. Robot. Res..

[19] Lang A.H., Vora S., Caesar H., Zhou L., Yang J., Beijbom O. Pointpillars: Fast encoders for object detection from point clouds. Proceedings of the IEEE Conference on Computer Vision and Pattern Recognition.

[20] Liu X., Qi C.R., Guibas L.J. Flownet3d: Learning scene flow in 3d point clouds. Proceedings of the IEEE/CVF Conference on Computer Vision and Pattern Recognition.

[21] Königshof H., Salscheider N.O., Stiller C. (2019). Realtime 3d object detection for automated driving using stereo vision and semantic information. Proceedings of the 2019 IEEE Intelligent Transportation Systems Conference (ITSC).

[22] Bijelic M., Gruber T., Mannan F., Kraus F., Ritter W., Dietmayer K., Heide F. Seeing Through Fog Without Seeing Fog: Deep Multimodal Sensor Fusion in Unseen Adverse Weather. Proceedings of the IEEE/CVF Conference on Computer Vision and Pattern Recognition.

[23] Meyer G.P., Charland J., Hegde D., Laddha A., Vallespi-Gonzalez C. Sensor fusion for joint 3d object detection and semantic segmentation. Proceedings of the IEEE Conference on Computer Vision and Pattern Recognition Workshops.

[24] Meyer G.P., Laddha A., Kee E., Vallespi-Gonzalez C., Wellington C.K. Lasernet: An efficient probabilistic 3d object detector for autonomous driving. Proceedings of the IEEE Conference on Computer Vision and Pattern Recognition.

[25] Du X., Ang M.H., Karaman S., Rus D. (2018). A general pipeline for 3d detection of vehicles. Proceedings of the 2018 IEEE International Conference on Robotics and Automation (ICRA).

[26] Chen L.C., Papandreou G., Kokkinos I., Murphy K., Yuille A.L. (2018). Deeplab: Semantic image segmentation with deep convolutional nets, atrous convolution, and fully connected crfs. IEEE Trans. Pattern Anal. Mach. Intell..

[27] Chen L.C., Zhu Y., Papandreou G., Schroff F., Adam H. Encoder-decoder with atrous separable convolution for semantic image segmentation. Proceedings of the European Conference on Computer Vision (ECCV).

[28] Dai J., Qi H., Xiong Y., Li Y., Zhang G., Hu H., Wei Y. Deformable convolutional networks. Proceedings of the IEEE International Conference on Computer Vision.

[29] Zhao H., Shi J., Qi X., Wang X., Jia J. Pyramid scene parsing network. Proceedings of the IEEE Conference on Computer Vision and Pattern Recognition.

[30] Cordts M., Omran M., Ramos S., Rehfeld T., Enzweiler M., Benenson R., Franke U., Roth S., Schiele B. The cityscapes dataset for semantic urban scene understanding. Proceedings of the IEEE Conference on Computer Vision and Pattern Recognition.

[31] Neuhold G., Ollmann T., Rota Bulo S., Kontschieder P. The mapillary vistas dataset for semantic understanding of street scenes. Proceedings of the IEEE International Conference on Computer Vision.

[32] Lin T.Y., Maire M., Belongie S., Hays J., Perona P., Ramanan D., Dollár P., Zitnick C.L. (2014). Microsoft coco: Common objects in context. Proceedings of the European Conference on Computer Vision.

[33] Romera E., Alvarez J.M., Bergasa L.M., Arroyo R. (2018). ERFNet: Efficient Residual Factorized ConvNet for Real-Time Semantic Segmentation. IEEE Trans. Intell. Transp. Syst..

[34] Lin G., Milan A., Shen C., Reid I. Refinenet: Multi-path refinement networks for high-resolution semantic segmentation. Proceedings of the IEEE Conference on Computer Vision and Pattern Recognition (CVPR).

[35] He K., Gkioxari G., Dollár P., Girshick R. Mask r-cnn. Proceedings of the IEEE International Conference on Computer Vision.

[36] Ren S., He K., Girshick R., Sun J. (2015). Faster r-cnn: Towards real-time object detection with region proposal networks. Adv. Neural Inf. Process. Syst..

[37] Fu C.Y., Shvets M., Berg A.C. (2019). RetinaMask: Learning to predict masks improves state-of-the-art single-shot detection for free. arXiv.

[38] Bolya D., Zhou C., Xiao F., Lee Y.J. YOLACT: Real-time instance segmentation. Proceedings of the IEEE International Conference on Computer Vision.

[39] Kendall A., Gal Y., Cipolla R. Multi-task learning using uncertainty to weigh losses for scene geometry and semantics. Proceedings of the IEEE Conference on Computer Vision and Pattern Recognition.

[40] Neven D., Brabandere B.D., Proesmans M., Gool L.V. Instance segmentation by jointly optimizing spatial embeddings and clustering bandwidth. Proceedings of the IEEE Conference on Computer Vision and Pattern Recognition.

[41] Gao N., Shan Y., Wang Y., Zhao X., Yu Y., Yang M., Huang K. Ssap: Single-shot instance segmentation with affinity pyramid. Proceedings of the IEEE International Conference on Computer Vision.

[42] Kirillov A., Levinkov E., Andres B., Savchynskyy B., Rother C. Instancecut: From edges to instances with multicut. Proceedings of the IEEE Conference on Computer Vision and Pattern Recognition.

[43] Liang J., Homayounfar N., Ma W.C., Xiong Y., Hu R., Urtasun R. Polytransform: Deep polygon transformer for instance segmentation. Proceedings of the IEEE/CVF Conference on Computer Vision and Pattern Recognition.

[44] Peng S., Jiang W., Pi H., Li X., Bao H., Zhou X. Deep Snake for Real-Time Instance Segmentation. Proceedings of the IEEE/CVF Conference on Computer Vision and Pattern Recognition.

[45] TensorRT. https://developer.nvidia.com/tensorrt.

[46] Moravec J., Šára R. Robust maximum-likelihood on-line LiDAR-to-camera calibration monitoring and refinement. Proceedings of the Computer Vision Winter Workshop.

[47] Kukelova Z., Heller J., Bujnak M., Fitzgibbon A., Pajdla T. Efficient solution to the epipolar geometry for radially distorted cameras. Proceedings of the IEEE International Conference on Computer Vision.

[48] Goga S.E.C., Nedevschi S. (2018). Fusing semantic labeled camera images and 3D LiDAR data for the detection of urban curbs. Proceedings of the 2018 IEEE 14th International Conference on Intelligent Computer Communication and Processing (ICCP).

[49] Schaupp L., Pfreundschuh P., Bürki M., Cadena C., Siegwart R., Nieto J. (2020). MOZARD: Multi-Modal Localization for Autonomous Vehicles in Urban Outdoor Environments. Proceedings of the 2020 IEEE/RSJ International Conference on Intelligent Robots and Systems (IROS).

[50] Florea H., Varga R., Nedevschi S. (2018). Environment Perception Architecture using Images and 3D Data. Proceedings of the 2018 IEEE 14th International Conference on Intelligent Computer Communication and Processing (ICCP).

[51] Arsigny V., Commowick O., Ayache N., Pennec X. (2009). A fast and log-euclidean polyaffine framework for locally linear registration. J. Math. Imaging Vis..

[52] Petrovai A., Nedevschi S. (2022). Semantic Cameras for 360-Degree Environment Perception in Automated Urban Driving. IEEE Trans. Intell. Transp. Syst..

[53] Costea A.D., Petrovai A., Nedevschi S. Fusion Scheme for Semantic and Instance-level Segmentation. Proceedings of the International Conference on Intelligent Transportation Systems (ITSC).

[54] Oniga F., Nedevschi S. (2018). A Fast Ransac Based Approach for Computing the Orientation of Obstacles in Traffic Scenes. Proceedings of the 2018 IEEE 14th International Conference on Intelligent Computer Communication and Processing (ICCP).

